# Electronic cigarettes and nicotine dependence: evolving products, evolving problems

**DOI:** 10.1186/s12916-015-0355-y

**Published:** 2015-05-21

**Authors:** Caroline O Cobb, Peter S Hendricks, Thomas Eissenberg

**Affiliations:** Department of Psychology, Center for the Study of Tobacco Products, Virginia Commonwealth University, 1112 East Clay Street, Suite B-08, Box 980205, Richmond, VA 23298 USA; Department of Health Behavior, School of Public Health, University of Alabama at Birmingham, 227L Ryals Public Health Building, 1665 University Boulevard, Birmingham, AL 35294 USA

**Keywords:** Dependence, Electronic cigarettes, Nicotine, Non-smokers, Smokers

## Abstract

Electronic cigarettes (ECIGs) use an electric heater to aerosolize a liquid that usually contains propylene glycol, vegetable glycerin, flavorants, and the dependence-producing drug nicotine. ECIG-induced nicotine dependence has become an important concern, as some ECIGs deliver very little nicotine while some may exceed the nicotine delivery profile of a tobacco cigarette. This variability is relevant to tobacco cigarette smokers who try to switch to ECIGs. Products with very low nicotine delivery may not substitute for tobacco cigarettes, so that ECIG use is accompanied by little reduced risk of cigarette-caused disease. Products with very high nicotine delivery may make quitting ECIGs particularly difficult should users decide to try. For non-smokers, the wide variability of ECIGs on the market is especially troublesome: low nicotine products may lead them to initiate nicotine self-administration and progress to higher dosing ECIGs or other products, and those that deliver more nicotine may produce nicotine dependence where it was not otherwise present. External regulatory action, guided by strong science, may be required to ensure that population-level nicotine dependence does not rise.

## Background

Electronic cigarettes (ECIGs) use an electric heater to aerosolize a liquid that usually contains some combination of propylene glycol, vegetable glycerin, flavorants, and nicotine. Nicotine is a mild psychomotor stimulant that supports repeated self-administration as well as the development of drug dependence, a neurobiological adaptation to repeated drug exposure that is manifested behaviorally by compulsive drug self-administration, an aversive withdrawal syndrome upon cessation, and an inability to quit despite a desire to do so and repeated cessation attempts [1,2]. There is an ongoing and lively debate regarding the potential for ECIGs to influence individual and public health for better [3,4] or for worse [5-7]. This debate frequently highlights the effects that ECIGs may have on cigarette smoking cessation and initiation, and in those contexts has touched on the impact of ECIGs on maintaining nicotine dependence where it already exists (i.e., in current cigarette smokers) or developing it where it does not (i.e., in never smokers or former smokers). The issue of ECIGs and nicotine dependence has become increasingly important in line with the rapid development of nicotine delivery methods, with earlier models largely being ineffective [8,9], and later models delivering nicotine to the user’s bloodstream much in the same manner as the tobacco cigarette [10] – a product optimally designed to increase the likelihood of chronic use or dependence [11]. If this evolution continues, ECIGs with a nicotine delivery profile that exceeds that of a tobacco cigarette may soon be commonplace [12]. The availability of such products may have profound effects for people who currently smoke tobacco cigarettes, as well as those who do not.

### Current smokers

For current tobacco cigarette smokers much has been written about the potential benefits of ECIGs [13,14]. These individuals are almost certainly already dependent on cigarette-delivered nicotine, and self-administer it via toxicant-laden tobacco smoke that causes a variety of lethal disorders including cancer, cardiovascular disease, and pulmonary disease [11]. The potential benefit of ECIGs for this population is that nicotine can be self-administered in an aerosol that contains far fewer tobacco toxicants at lower levels than those found in tobacco smoke and thus may present a reduced health risk [15], although the long-term health outcomes of chronic ECIG use are unknown. For this potential of ECIGs to be realized to its fullest extent, inhalation of toxic tobacco smoke must cease almost completely [16,17], meaning that tobacco cigarette smokers must use ECIGs as a total or near-total substitute for cigarette smoking. Recent data indicate that ECIG use acts as a substitute for cigarette smoking for some individuals, but not for others [18]. Total substitution for the majority of smokers may be more likely when ECIGs reliably approximate the nicotine delivery profile of a tobacco cigarette the first time and every time they are used. If ECIG producers are unwilling to ensure that their products perform in this manner, empirically-based ECIG regulation may have an important role to play in ensuring safe and reliable nicotine delivery.

Importantly, ECIGs may continue to evolve such that they exceed the nicotine delivery profile of a tobacco cigarette. For ECIG producers, providing ever more nicotine to already nicotine-dependent individuals may be seen as a business growth opportunity: what better way to escalate profits than to induce customers to use more product compulsively? Obviously, if long-term ECIG use is, at some point in the future, demonstrated to cause adverse health consequences, this seeming ‘growth opportunity’ could be a public health disaster. Less obviously, even if ECIG use causes no adverse health consequences, there are still individual and societal harms associated with ever increasing levels of drug dependence. Dependent individuals can spend inordinate resources on drug seeking and self-administration, and prioritize these behaviors over occupational, familial, and other obligations [2]. Consider that pathological internet use, computer game-playing, and gambling represent problematic behaviors on the addictive spectrum that are associated with few direct negative physical health effects, yet important psychological and behavioral sequelae [19-21]. Put simply, there is no clear public health justification for the ready availability of ECIGs that exceed the nicotine delivery profile of tobacco cigarettes. There is already evidence that ECIG use maintains some level of nicotine dependence [22,23], even with products that likely do not deliver nicotine efficiently. If excessive nicotine delivery renders ECIG users even more dependent and thus even more unable to quit should they eventually decide to try, this additional loss of control itself could become a legitimate public health concern. If ECIG producers are unwilling to ensure that their products do not exceed the nicotine delivery profile of tobacco cigarettes, empirically-based ECIG regulation may have an important role to play in ensuring safe and reliable nicotine delivery that does not exceed this profile.

### Current non-smokers

Non-smokers include never smokers and former smokers, few of whom currently are nicotine dependent. These individuals are already at risk for ECIG-initiated nicotine dependence due to marketing methods that may target them [24,25], nicotine-containing liquids that mimic the flavors of highly palatable foods and drinks [25,26], and relatively unrestricted ECIG access [27]. Survey data suggest that at least some non-smokers are already experimenting with ECIGs [28-31]. The extent to which this experimentation will become compulsive use is unclear. If it does, all of the arguments above become more compelling. In addition to the risks associated with ECIGs that deliver nicotine at a rate above a tobacco cigarette, ECIGs that deliver low levels of nicotine may function as the so-called ‘starter products’ common in the smokeless tobacco arena [32]. These starter products allow nicotine-naïve users to self-administer low doses of nicotine without experiencing drug-mediated adverse side effects, and then, as tolerance develops, they can ‘graduate’ to products that deliver increasing doses of the drug [32]. Thus, ECIGs that deliver little nicotine might start nicotine-naïve users on the trajectory to compulsive nicotine use, whereas products that deliver even more nicotine than a tobacco cigarette have the potential to make ECIG cessation even more difficult than smoking cessation. A similar line of reasoning follows for former smokers, who not only risk a return to nicotine dependence via ECIGs, but also the possibility of relapse to their previously-preferred nicotine self-administration method, the lethal tobacco cigarette. If ECIG producers are unwilling to act so that their products do not lead never-smokers and former smokers into compulsive nicotine use, empirically-based ECIG regulation may have an important role to play in avoiding this outcome.

## Conclusions

The evolution of the ECIG from a class of products that failed to deliver nicotine to one that has the potential to exceed the nicotine delivery of a tobacco cigarette is a concern for all. This concern is not based on some ideological or moral position regarding drug dependence [4], but rather on an understanding of the financial, behavioral, and social ramifications of compulsive drug use. As others have suggested [4,13,14], ECIG use well may be a method for achieving significant decreases in the disability, disease, and death associated with combustible tobacco use worldwide. Achieving these decreases may require ECIGs that approach the nicotine delivery profile of a tobacco cigarette, but likely do not require ECIGs that exceed that profile. In addition, these decreases in cigarette-caused morbidity and mortality must not be accompanied by an increase in compulsive nicotine use among those who do not currently use the drug. A profit-minded ECIG industry may require external regulatory force, guided by strong science, to ensure that population-level nicotine dependence does not rise. Relevant targets for further research and potential regulatory intervention include product characteristics [33] and nicotine flux [34,35], as well as product advertising and access [36]. In addition, this discussion has focused exclusively on nicotine; regulation can also limit user exposure to other ECIG toxicants, including those contained in the liquid [37,38] as well as those produced when the liquid is heated [37,39,40].
